# Adaptation and validation of a Spanish version of the treatment burden questionnaire in patients with multiple sclerosis

**DOI:** 10.1186/s12883-019-1441-0

**Published:** 2019-08-27

**Authors:** María Célica Ysrraelit, Marcela Paula Fiol, Fernando Vazquez Peña, Sandra Vanotti, Sergio Adrián Terrasa, Viet-Thi Tran, Victor M. Montori, Jorge Correale

**Affiliations:** 1 Department of Neurology, FLENI, Buenos Aires, Argentina; 2 Hospital Italiano de Buenos Aires (HIBA), Buenos Aires, Argentina; 3 Multiple Sclerosis Clinic, INEBA - Neurosciences Institute of Buenos Aires, Buenos Aires, Argentina; 4 Centre of Research in Epidemiology and Statistics (CRESS – UMR 1153), Paris, France; 5 Knowledge and Evaluation Research Unit, Mayo Clinic, Rochester, MN 55905 USA

**Keywords:** Treatment burden, QoL, Patient-reported-outcome, Questionnaires, Multiple sclerosis, Minimally disruptive medicine

## Abstract

**Background:**

The Treatment Burden Questionnaire (TBQ) is a self-reported measure of the effect of treatment workload on patient wellbeing. We sought to validate the TBQ in Spanish and use it to estimate the burden of treatment in Argentinian patients with multiple sclerosis (MS).

**Methods:**

The TBQ was forward-backward translated into Spanish. Two focus groups and 25 semi-structured interviews focused on wording and possible item exclusion. Validation was performed in 2 steps. First, 162 patients across a range of MS severity completed the questionnaire. Confirmatory factor analysis assessed the dimensional structure of the TBQ. Construct validity was assessed by studying correlations with fatigue and quality of life (QoL). Then, in a second cohort of 171 patients, we evaluated the association between TBQ scores and patients’ sex, age, education level, employment status, type of MS, disease duration, comorbidities, EDSS, pharmacological treatment and medication adherence.

**Results:**

The questionnaire presented a 3-factor structure in which burden was related to pharmacological treatment; comprehensive health assistance; and psycho-social-economic context. Composite reliability was > 0.8 for all factors. TBQ showed positive correlation with fatigue (r_s_ = 0.467, *p* = 0.006), negative correlation with QoL (r_s_ − 0.446, *p* = 0.009). For the second cohort, total TBQ score was 43 (SD 29). Lowest scores were observed on self-monitoring (0.53, SD 1.3) and highest for administrative load (4.2, SD 3.4). Inverse association was found between the TBQ score and medication adherence (r 0.243 *p* = 0.001). TBQ scores also correlated with daily patient pill/injection requirements (r 0.175 *p* = 0.020). Individuals receiving injectable treatment scored higher than patients on oral drugs (total TBQ 51 (SD 32) vs 39 (SD 27) *p* = 0.002).

**Conclusions:**

The TBQ in Spanish is a reliable instrument and showed adequate correlation with QoL and adherence scales in MS patients. TBQ may benefit health resources allocation and provide tailor therapeutic interventions to construct a minimally disruptive care.

**Electronic supplementary material:**

The online version of this article (10.1186/s12883-019-1441-0) contains supplementary material, which is available to authorized users.

## Background

The burden of treatment refers to efforts patients need to make to access and use healthcare and to adhere to self-care activities (e.g., medication adherence, self-monitoring, clinic visits, lifestyle changes) and to the negative effect these efforts have on patient wellbeing. This ‘work’ of being a patient [1, 2] requires significant investments in time, money, and physical, emotional and cognitive energy, ultimately decreasing quality of life (QoL) [3, 4].

Multiple sclerosis (MS) is a complex chronic condition often affecting individuals at the most productive stage of life. In many countries, it is the leading cause of non-traumatic disability in young adults. MS patients experience many different physical symptoms such as restricted mobility, visual deficits, balance disorders, sexual and urinary dysfunction, as well as chronic pain, among others. In addition, cognitive impairment and fatigue are present in a significant number of subjects. All of these health issues make disease burden very high in MS [5–7]. Most patients require multiple clinic visits and treatments – pharmacological, physical therapy, cognitive neurorehabilitation – to manage the disease, avoid disability and maintain their quality of life. All these needs further increase the burden of comprehensive treatment. Some MS treatments involve self-administered injections or infusions, which can be extremely challenging for some patients to carry out. In one study, almost 15% of MS patients were unable to self-administer the full dose of a subcutaneously injection [8].

Patient self-assessment of their condition or of the effects of treatment varies significantly between cultures. Differences relating to concepts of health and illness, as well as of socially desirable effects are key issues when using patient-reported outcome measures in the evaluation of medicinal products, a concern shared by both EMA and FDA [9, 10]. Several questionnaires most often developed in English, have been translated for use in other countries, and regulatory authorities are rightfully concerned about cross-cultural validity when attempting to measure the same concepts. The Treatment Burden Questionnaire (TBQ) is an instrument designed to assess the burden of treatment for different medical conditions and contexts. The instrument was first developed in a sample of patients with one or more chronic conditions in France [4], and then translated to English and adapted for use within other health systems [1]. It includes 15 items rated on a Likert scale ranging from 0 (not a problem), to 10 (significant problem). Item scores are added together to generate a total global score, ranging from 0 to 150.

Our objective was, first, to validate the TBQ scale in Spanish in a population of MS patients; and, second, to identify specific clinical and demographic factors that were associated with patients’ burden of treatment. This is important for better health resource allocation and to individualize and tailor therapeutic interventions.

## Methods

### Population

This study was carried out in patients diagnosed with MS attending the Neurology clinic at FLENI in Buenos Aires, Argentina. Inclusion criteria were: diagnosis of MS according to 2010 revisions to the McDonald criteria [11], age 18–99 years, native Spanish speakers, and ability to provide informed consent. Exclusion criteria included physical or psychological inability to complete tests and presence of relapse and/or administration of corticosteroids 4 weeks prior to assessment.

The protocol was approved by the local Ethical Committee and all participants gave written informed consent.

### Validation of the instrument in a first cohort of patients

A multistep approach was used [12] to validate: 1) content 2) language and 3) psychometric properties [12].

#### Content validation

We studied the content validity of the TBQ for MS patients (i.e. whether the TBQ was relevant for MS patients) by answering the questions: 1) Does the TBQ adequately reflect the difficulties presented to patients with MS treatment? and 2) Is there any relevant aspect applicable to the local environment that is not taken into account by the instrument? To answer these questions, a review of studies about burden of treatment in Latin America was performed. The following databases were queried to identify relevant articles: 1) Medline (Pubmed), 2) EMBASE 3) Latin American and Caribbean Center on Health Sciences Information (BIREME) 4) Scientific Electronic Library on Line (SciELO) Then, two focus groups were organized: patients who consulted on an outpatient basis at the neuroimmunology department in the last month were selected to represent different clinical forms of MS (relapsing-remitting, secondary-progressive and/or primary-progressive), as well as different degrees of disability, forms of treatment and disease duration. During interviews conducted with trigger questions, domains proposed in the TBQ instrument were explored and degree of agreement with perceptions of local patients evaluated. Additional domains not included in the original version were also assessed.

#### Language validation

The TBQ was translated into Spanish applying a forward and backward translation procedure. The questionnaire was first translated into Argentine Spanish. Then two translations into English were carried out by native English speakers living in Argentina, both of which were consolidated into an initial Spanish version. The first consolidated version was then translated back into English by a different native English speaker, and this final version sent to the original author of the questionnaire and to the team that translated the original French language questionnaire into English. After approval by the original authors, a reading test was performed in 25 semi-structured interviews where wording, level of understanding and possible exclusion of items were assessed.

#### Psychometric validation

In a cohort of 162 heterogeneous MS patients, the dimensional structure of the TBQ was studied with confirmatory factor analysis (CFA), using the Unweighted Least Squares estimator and the Lisrel program. Uni, bi, or tri- dimensional structures were analyzed from a conceptual point of view. In the case of two-dimensional structures, one factor was considered to be treatment burden related to medication, and the other, the remaining items combined. In the case of three-dimensional structures, items were grouped according to burden of treatment related to (1) medication, (2) healthcare, and (3) psycho-socioeconomic context. In addition, a subgroup of 33 patients also completed the following tests: Expanded Disability Status Scale (EDSS) [13], Multiple Sclerosis Functional Composite [14], Symbol Digit Modalities Test [15], Brief International Cognitive Assessment for Multiple Sclerosis [16], Multiple Sclerosis International Quality of Life Questionnaire (MSQoL) [17], Fatigue Severity Scale (FSS) [18], MOS social support scale [19], Beck Depression Inventory [20] and Hospital Anxiety and Depression Scale [21]. We studied the correlation between the TBQ and those scales using Spearman’s (r_s_) and Pearson’s correlation coefficients. We expected a negative correlation between burden of treatment (measured by TBQ) and QoL (measured by MSQoL); and a positive correlation between TBQ scores and fatigue (measured by FSS).

### Assessment of the relationships between burden of treatment and MS patients’ characteristics

In a second cohort of 171 MS patients [111 (65%) females, mean age 42 (SD 10)] who did not participate in the first phase of the study, we investigated the association between burden of treatment, measured using the TBQ and clinical and demographic factors (sex, age, type of MS, disease duration, education level, employment status, presence of comorbidities, EDSS, pharmacological treatment and medication adherence. Adherence was assessed by the Morisky Green Levine Medication Adherence Scale (MGL) [22]. Associations were studied using Pearson’s coefficient [23]. Reliability was assessed calculating composite reliability.

## Results

### Validation of the instrument in a first cohort of patients

Content of the TBQ was tested during two focus groups and 25 semi-structured interviews in 38 MS patients (25 females), aged 43 (SD 7.9) with a mean EDSS of 2.1 (range 0–8). Patients expressed difficulties with exams, not in relation to frequency, time spent on them or associated inconveniences, but to the uncertainty generated over test results to know whether prescribed treatments were working:

*“I don’t care if I’m in the MRI machine for two hours, but the days following the study, while waiting to see my doctor again, are terrible for me”*, said a 35 year-old female patient who has been living with MS for 10 years.

Patients with MS usually require an MRI once or twice a year to assess disease activity and identify suboptimal response to treatment. This is true not only in MS but also in other chronic diseases, where evidence shows a central and disruptive role for both uncertainty over potential threats in subclinical conditions, and for clinical anxiety, that may impact patient health [24]. We therefore added a new question to the Spanish version of the TBQ to explore this item, as follows: *‘How would you rate the anxiety generated by uncertainty over results of medical evaluations or tests ordered to check if your treatment is working?*”. As a result, the maximum TBQ score was now 160 (instead of 150). Final English version of the questionnaire is available in Additional file 1.

In contrast, items related to self-monitoring, dietary changes and recommendations to practice physical activity generated poor factorial load. Given that all three items mentioned provided important information for patients with comorbidities, these were kept in the questionnaire, but were excluded from the factor analysis. Correlation between both versions was > 0.9 and considered equivalent [25].

**Fig. 1:**
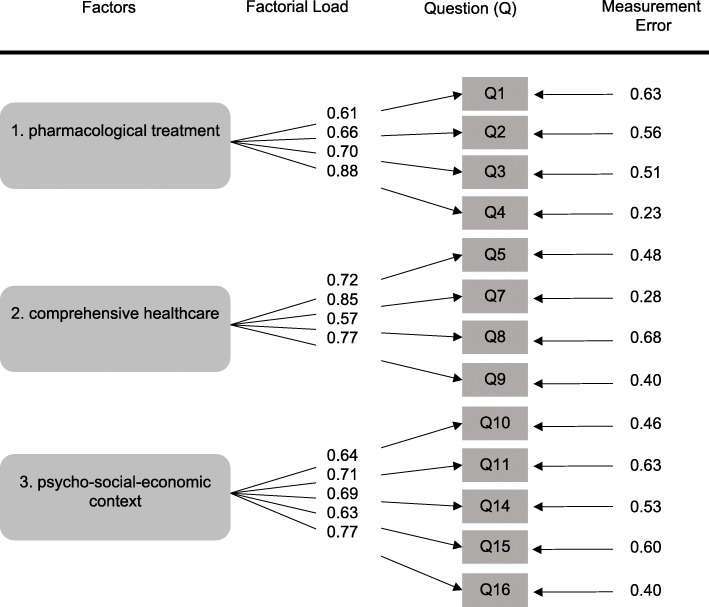
Short Title Confirmatory Factor Analysis. Three factors model: standard values. Detailed legend Standard values of 3 factor model. Q refers to item number on the Treatment Burden Questionnaire as detailed in Table 3. Questions 6, 12 and 13 were excluded because their factorial load was low

Confirmatory factor analysis showed better adjustment as the number of factors included increased from 1 to 3 (Table 1 and Fig. 1).

**Table 1:** Factorial Validity of Treatment Burden Questionnaire

	CFI (> 0.9)	NNFI (> 0.9)	RMSEA (< 0.08)	RMRs (< 0.08)	AGFI (> 0.9)
Unifactorial	0.89	0.87	0.13	0.12	0.90
Two Factors	0.96	0.95	0.083	0.077	0.96
Three Factors	0.98	0.97	0.063	0.068	0.96

Confirmatory Factor Analysis determining the dimensional structure of the questionnaire by use of one, two or three factors. *CFI* Comparative Fit Index, *NNFI* (Non) NormedFit Index, *RMSEA* Root Mean Square Error of Approximation RMRs Standardized Root Mean Square Residual, *AGFI* (Adjusted) Goodness of Fit

Composite reliability > 0.8 for all three factors. Validity of the external criterion was optimal according to reference values (25). Construct validity showed significant correlation between the TBQ and presence of fatigue measured by FSS [18] (r_s_ = 0.467, *p* = 0.006), as well as with QoL, measured by MSQoL [17] (r_s_ − 0.446, *p* = 0.009). No correlation with EDSS or other scales was observed.

### Assessment of the relationships between burden of treatment and MS patients’ characteristics

After the initial validation of the questionnaire, an additional cohort of 171 MS patients (Table 2) were surveyed with the TBQ (Table 3). Mean total TBQ was 43 (SD 29). The lowest mean (SD) score was for the item related to self-monitoring (0.5, SD 1.3) and the highest for administrative load (4.2, SD 3.4). Interestingly, the new question we added about anxiety over uncertain test results to evaluate response to treatment was considered the second most important factor contributing to burden of treatment (TBQ score = 3.7, SD 3.5). This is not comparable with previous versions of the TBQ in French or English as they had 13 or 15 questions respectively [1, 4].

**Table 2:** Clinical and Demographical Data of the second cohort of MS patients

Total n	171
Age, mean (SD)	42 (10) years
Women, n (%)	111 (65)
Education, mean (SD)	17 (3.6) years
Employment, n (%)	
Full time	103 (61)
Part time	30 (18)
Unemployed	29 (17)
Retired due to disability	8 (5)
Type of MS, n (%)	
RRMS	133 (78)
PPMS	11 (7)
SPMS	11 (7)
CIS	4 (2)
Unknown	11 (7)
Disease Duration, mean (SD)	9.2 (7) years
EDSS mean (range)	1.86 (0–9)
Comorbidities, n (%)	
Smoking	24 (14)
BMI > 30	24 (14)
Diabetes	5 (3)
Hypertension	14 (9)
Dyslipidaemia	37 (24)
Other autoimmune disease	24 (16)
Disease-Modifying Treatment, n (%)	
Injectable	58 (34)
Oral	73 (42)
Monoclonal Antibodies	15 (9)
None	21 (12)
Total number of pills or injections per patient per day, mean (SD)	2.7 (2.1)

**Table 3:** TBQ final questionnaire scores per item

TBQ Item	Score, mean (SD)
1. The taste, shape or size of your tablets and/or the annoyances by your injections	2.6 (3.2)
2. The numbers of times you should take your medication daily	1.8 (2.3)
3. The efforts you make not to forget to take your medications	2.5 (2.9)
4. The necessary precautions when taking your medication	2.1 (2.8)
5. Lab Tests and other exams: frequency, time spent and associated nuisances or inconveniences	2.8 (2.6)
6. Self-monitoring: frequency, time spent and inconveniences	0.5 (1.3)
7. Doctor visits and other appointments: frequency, time spent for these visits and difficulties findings healthcare providers	2.7 (2.8)
8. Difficulties in relationships with healthcare providers	1.6 (2.6)
9. Arranging medical appointments and/or transportation and reorganizing schedules around these appointments	3.3 (3.1)
10. Administrative burden related to healthcare	4.2 (3.4)
11. Financial burden associated with healthcare	3.2 (3.2)
12. Burden related to dietary changes	2.1 (2.8)
13. Burden related to doctors recommendations to practice physical activity	3.0 (3.2)
14. Impact of healthcare on relationships with others	3.0 (3.4)
15. Anxiety generated by uncertainty over results of medical checkups and complementary studies to know if treatment is working	3.6 (3.5)
16. The need for medical healthcare on a regular basis reminds me of my health problem	2.9 (3.2)

Again, we found a significant and inverse correlation between TBQ and QoL (r_s_ − 0.446, *p* = 0.009) and a positive correlation with fatigue (r_s_ 0.467, *p* = 0.006). In addition, in this cohort we found an inverse association between TBQ global score and medication adherence: the greater the treatment burden, the lower the adherence to treatment (r 0.243 *p* = 0.001). TBQ scores also showed weak correlation with number of pills or injections (r 0.175 *p* = 0.020). Number of comorbidities correlated with daily number of pills or injections (r 0.240 *p* = 0.02) but not with TBQ. There were no differences in TBQ scores across men and women, type of MS, disease duration or employment status. Patients receiving injectable therapies had significantly higher burden of treatment than patients taking oral drugs (TBQ total injectable 51 (SD 32) vs oral 39 (SD 27) *p* = 0.002).

## Discussion

TBQ is the first reliable tool assessing treatment burden across multiple conditions and treatments [4]. We adapted a Spanish version of this instrument for patients with MS, a chronic complex neurological condition. During the process, we added a new item to the Spanish TBQ, related to dealing with uncertain test results, not included in the original English version. As expected, we found direct correlation between treatment burden and QoL (i.e., patients with higher burden had poorer QoL) and not with other measures of burden of illness.

We found a correlation between TBQ scores and fatigue. Fatigue is considered by MS patients to be one of the main causes of impaired quality of life, independent of depression or disability [26, 27] and is the most commonly reported symptom [28]. More than 25% of all patients, state fatigue as their most disabling symptom [29]. Patients overwhelmed by treatment demands may be fatigued by these demands either because of excessive workload or reduced capacity, including reduced capacity because of burden of illness.

We found significant differences in TB in patients receiving oral versus injectable therapies. This has been previously described [30, 31] and is an additional factor that must be taken into account when choosing specific disease-modifying treatments for the individual patient. We also detected significant correlation between TBQ scores and medication adherence. This correlation was previously reported [1, 4]. Non-adherence in MS is further related to suboptimal response to treatment [32], including disease relapse [32], decreased QoL, and need for more expensive healthcare, such as increased number of emergency department visits and hospitalizations [8, 33].

Other instruments to measure TB have been recently developed: the Patient Experience with Treatment and Self- management (PETS) [34] and the Medication-Related Burden Quality of Life (MRB-QoL) tool [35]. We chose TBQ because it is a concise, user-friendly, and comprehensive measurement, whose scores can be reliably compared across future studies with potential application for research and in clinical practice. Furthermore, the evaluation of the psycho-social-economic context is a critical part of patient-centered care in order to promote optimal medical outcomes and psychosocial well-being, in patients with chronic illnesses like MS.

In the literature, many factors are considered to impact treatment load. Sav et al. have applied the term “antecedents” to describe them, and include not only those linked to disease, for example elevated number of medications or dosage forms (oral versus injections), but also individual patient characteristics including: age, sex, employment status, family support and engagement, presence of comorbidities as well as patient/health provider relationship [36, 37]. Treatment burden is thought to result from imbalance between patient “workload” and patient “capacity”. Patients with few demands but low capacity may experience more burden whereas those with many demands but high capacity, may not. Examples of patient capacity include personal attributes and skills, physical and cognitive abilities, and social and financial support [38–40].

The future of chronic care encompasses technological innovations that may help to diminish treatment burden. For example, mobile phones represent a platform increasing access to care that can help improve communication between patients and healthcare providers, thereby reducing burden associated with travel and administrative issues, which in this study population were the most significant factors associated with treatment burden [41].

A first step towards lessening treatment burden is the development of assessment tools like the TBQ. Patient experience is a concept that has been gaining attention as an element of quality healthcare. Recently, patient-reported outcomes (PROs) and patient-reported outcome measures (PROMs) have been recognized as important to improve healthcare services, clinical practice and outcome research [42]. Similarly, TB can serve as a patient reported indicator of the effect that patient work has on social, physical and psychological functioning of patients with chronic conditions [43].

Our study has some limitations. We conducted a cross-sectional study that drew mostly privately insured patients with high educational level from one medical center. This limits the applicability of our findings to patients with lower socio-economic status cared and receiving care in other health systems in whom the financial burden of treatment may dominate the picture. Longitudinal studies may be needed to better understand how burden of treatment changes with fluctuations of MS activity, emergence of comorbidity, and changes in treatment intensity over time. Finally, depending on patient family structure or social environment, patients may share the burden of treatment with informal caregivers; the burden of treatment reported by patients in these cases may underestimate the total treatment burden carried by patients and caregivers.

The concept of moving towards a future of ‘minimally disruptive medicine’ has been proposed, where patient care emphasizes individual preferences, takes multimorbidity into account and tries to reduce workload for patients and caregivers [2]. Patient-centered frameworks, such as minimally disruptive medicine, aim to minimize TB by optimizing the workload necessary to achieve patient goals, while boosting capacity [43]. To do this, we need to provide coordinated care, centered on the person and not on the disease. Simple questions from treating physicians could be the first step in this direction, for example: “Can you really do what I am asking you to do?” [44].

## Conclusion

We have produced a valid Spanish version of the TBQ and demonstrated its value in characterizing for the first time the burden of treatment in patients with MS in Latin America. Our results may help physicians better understand and identify patients who are overwhelmed by the complexity of their treatment, and highlights the need to change the current paradigm toward minimally disruptive medicine [2].

## Additional file

Additional file 1: Final English language version of the Treatment Burden Questionnaire developed specifically for this study and complete references of all other questionnaires used in this study. (DOCX 17 kb)

## Data Availability

The datasets used and/or analyzed during the current study are available from the corresponding author on reasonable request.
