# Supplementary material for: Adaptation and validation of a Spanish version of the treatment burden questionnaire in patients with multiple sclerosis
Source: BMC Neurol. 2019 Aug 27;19:209. doi: 10.1186/s12883-019-1441-0 (PMC6710872; doi:10.1186/s12883-019-1441-0)
Supplement: Supplementary file 1 — Final English language version of the Treatment Burden Questionnaire developed specifically for this study and complete references of all other questionnaires used in this study. (DOCX 17 kb) [file 12883_2019_1441_MOESM1_ESM.docx]

Supplementary files

In the present work we performed a Spanish validation of the original Treatment Burden Questionnaire. Final Complete Questionarie include the following statement and 16 questions:

Instructions: Please read each of the following statements carefully. They evaluate the effort that can represent taking care of your illness. Assign each one a score

from 0 to 10 where 0 means NO EFFORT and 10 MAXIMUM EFFORT. Once you choose your answer, mark it in the corresponding box with an X.

Thinking about the treatment of your MS, how much effort did it represent ...?

| TBQ Items | 0 | 1 | 2 | 3 | 4 | 5 | 6 | 7 | 8 | 9 | 10 |
| --- | --- | --- | --- | --- | --- | --- | --- | --- | --- | --- | --- |
| **The taste, shape or size of your tablets and/or the annoyances by your injections** ( e.g pain, bleeding, bruising or scars) |  |  |  |  |  |  |  |  |  |  |  |
| **The numbers of times** you should take your medication daily |  |  |  |  |  |  |  |  |  |  |  |
| The efforts you make **not to forget to take your medications** ( e.g managing your treatment when you are away from home, preparing and using pillboxes |  |  |  |  |  |  |  |  |  |  |  |
| The **necessary precautions when taking your medication** ( e.g taking them at specific times of the day or meals, not being able to do certains things after medications such driving or lying down) |  |  |  |  |  |  |  |  |  |  |  |
| **Lab Tests and other exams** ( e.g blood tests or radiology): frequency, time spent and associated nuisances or inconveniences |  |  |  |  |  |  |  |  |  |  |  |
| **Self monitoring** ( e.g taking your blood pressure or checking your blood sugar): frequency, time spent and nuisances or inconveniences |  |  |  |  |  |  |  |  |  |  |  |
| **Doctor visits and others appointments**: frequency, time spent for these visits and difficulties findings healthcare providers |  |  |  |  |  |  |  |  |  |  |  |
| The difficulties you could have in your **relationships with healthcare providers** ( e.g feeling not listening to enough or not taken seriously) |  |  |  |  |  |  |  |  |  |  |  |
| **Arranging medical appointments and/or transportation** ( doctors visits, labs test and other exams) and reorganizing your schedule around these appointments |  |  |  |  |  |  |  |  |  |  |  |
| The **administrative burden related to healthcare** ( e.g all you have to do for hospitalizations, insurance reimbursements and/or obtaining social services) |  |  |  |  |  |  |  |  |  |  |  |
| The **financial burden associated with your healthcare** ( e.g out-to-pocket expenses or expenses not covered by insurances) |  |  |  |  |  |  |  |  |  |  |  |
| The **burden related to dietary changes** ( e.g avoiding certains foods or alcohol, having to quit smoking) |  |  |  |  |  |  |  |  |  |  |  |
| The **burden related to doctors´recommendations to practice physical activity** ( e.g walking, jogging, swimming) |  |  |  |  |  |  |  |  |  |  |  |
| How does your healthcare impact your **relationships with others** ( e.g being dependent on others and feeling like a burden to them, being embarrassed to take your medication in public) |  |  |  |  |  |  |  |  |  |  |  |
| 15. Anxiety generated by uncertainty over results of medical checkups and complementary studies to know if treatment is working |  |  |  |  |  |  |  |  |  |  |  |
| **The need for medical healthcare on a regular basis reminds me of my health problem** |  |  |  |  |  |  |  |  |  |  |  |

Original questionnaire is available at

Tran V-T, Harrington M, Montori VM, Barnes C, Wicks P, Ravaud P. Adaptation and validation of the Treatment Burden Questionnaire (TBQ) in English using an internet platform. BMC Med [Internet]. 2014 Dec 2 [cited 2018 Sep 4];12(1):109. Available from: <http://www.ncbi.nlm.nih.gov/pubmed/24989988>

Additionally patients were evaluated with

1. Expanded Disability Status Scale. Available at Kurtzke JF. Rating neurologic impairment in multiple sclerosis: an expanded disability status scale (EDSS). Neurology [Internet]. 1983 Nov [cited 2019 Feb 6];33(11):1444–52. Available from: <http://www.ncbi.nlm.nih.gov/pubmed/6685237>
2. Multiple Sclerosis Functional Composite. Available at Cutter GR, Baier ML, Rudick RA, Cookfair DL, Fischer JS, Petkau J, et al. Development of a multiple sclerosis functional composite as a clinical trial outcome measure. Brain [Internet]. 1999 May [cited 2019 Feb 6];122 ( Pt 5):871–82. Available from: <http://www.ncbi.nlm.nih.gov/pubmed/10355672>
3. Symbol Digit Modalities Test . Available at: Benedict RH, DeLuca J, Phillips G, LaRocca N, Hudson LD, Rudick R, et al. Validity of the Symbol Digit Modalities Test as a cognition performance outcome measure for multiple sclerosis. Mult Scler J [Internet]. 2017 Apr 16 [cited 2019 Feb 6];23(5):721–33. Available from: <http://www.ncbi.nlm.nih.gov/pubmed/28206827>
4. Brief International Cognitive Assessment for Multiple Sclerosis (BICAMS). Available at: Langdon DW, Amato MP, Boringa J, Brochet B, Foley F, Fredrikson S, et al. Recommendations for a Brief International Cognitive Assessment for Multiple Sclerosis (BICAMS). Mult Scler [Internet]. 2012 Jun 21 [cited 2019 Feb 6];18(6):891–8. Available from: <http://journals.sagepub.com/doi/10.1177/1352458511431076>
5. Vickrey BG, Hays RD, Harooni R, Myers LW, Ellison GW. A health-related quality of life measure for multiple sclerosis. Qual Life Res [Internet]. 1995 Jun [cited 2019 Feb 6];4(3):187–206. Available from: <http://www.ncbi.nlm.nih.gov/pubmed/7613530>
6. Fatigue Severity Scale. Available at Krupp LB, LaRocca NG, Muir-Nash J, Steinberg AD. The fatigue severity scale. Application to patients with multiple sclerosis and systemic lupus erythematosus. Arch Neurol [Internet]. 1989 Oct [cited 2019 Feb 6];46(10):1121–3. Available from: <http://www.ncbi.nlm.nih.gov/pubmed/2803071>
7. MOS social support survey. Availabe from Sherbourne CD, Stewart AL. The MOS social support survey. Soc Sci Med [Internet]. 1991 [cited 2019 Feb 6];32(6):705–14. Available from: <http://www.ncbi.nlm.nih.gov/pubmed/2035047>
8. Beck Depression Inventory. BECK AT, WARD CH, MENDELSON M, MOCK J, ERBAUGH J. An inventory for measuring depression. Arch Gen Psychiatry [Internet]. 1961 Jun [cited 2019 Feb 6];4:561–71. Available from: <http://www.ncbi.nlm.nih.gov/pubmed/13688369>
9. The hospital anxiety and depression scale. Zigmond AS, Snaith RP. The hospital anxiety and depression scale. Acta Psychiatr Scand [Internet]. 1983 Jun [cited 2019 Feb 6];67(6):361–70. Available from: <http://www.ncbi.nlm.nih.gov/pubmed/6880820>
10. Morisky DE, Green LW, Levine DM. Concurrent and predictive validity of a self-reported measure of medication adherence. Med Care [Internet]. 1986 Jan [cited 2019 Feb 6];24(1):67–74. Available from: <http://www.ncbi.nlm.nih.gov/pubmed/3945130>
